# The Rice Phytochrome Genes, *PHYA* and *PHYB*, Have Synergistic Effects on Anther Development and Pollen Viability

**DOI:** 10.1038/s41598-017-06909-2

**Published:** 2017-07-25

**Authors:** Wei Sun, Xiao Hui Xu, Xingbo Lu, Lixia Xie, Bo Bai, Chongke Zheng, Hongwei Sun, Yanan He, Xian-zhi Xie

**Affiliations:** 1 Shandong Rice Research Institute, Shandong Academy of Agricultural Sciences, Ji’nan, 250100 China; 2 Institute of Plant Protection, Shandong Academy of Agricultural Sciences, Shandong Key Laboratory of Plant Virology, Ji’nan, 250100 China

## Abstract

Phytochromes are the main plant photoreceptors regulating multiple developmental processes. However, the regulatory network of phytochrome-mediated plant reproduction has remained largely unexplored. There are three phytochromes in rice, phyA, phyB and phyC. No changes in fertility are observed in the single mutants, whereas the seed-setting rate of the *phyA phyB* double mutant is significantly reduced. Histological and cytological analyses showed that the reduced fertility of the *phyA phyB* mutant was due to defects in both anther and pollen development. The four anther lobes in the *phyA phyB* mutant were developed at different stages with fewer pollen grains, most of which were aborted. At the mature stage, more than one lobe in the double mutant was just consisted of several cell layers. To identify genes involved in phytochrome-mediated anther development, anther transcriptomes of *phyA*, *phyB* and *phyA phyB* mutants were compared to that of wild-type rice respectively. Analysis of 2,241 double-mutant-specific differentially expressed transcripts revealed that the metabolic profiles, especially carbohydrate metabolism, were altered greatly, and heat-shock responses were activated in the double mutant. This study firstly provides valuable insight into the complex regulatory networks underlying phytochrome-mediated anther and pollen development in plants, and offers novel clues for hybrid rice breeding.

## Introduction

Rice (*Oryza sativa* L.), one of the most important crops in the world, has been extensively used as a monocot model crop in developmental biology studies^1^. Male fertility, an essential prerequisite for successful fertilization, depends on well-developed anthers and male gametophytes. On the basis of morphology, rice anther development has been divided into 14 stages, similar to that of *Arabidopsis* ^2^.

Forward and reverse genetic studies have identified a number of key regulators involved in male fertility control, including transcription factors, receptor kinases and hormones^3, 4^. Several rice genes that play important roles in meiosis have also been identified, such as *PAIR1* (*HOMOLOGOUS PAIRING ABERRATION IN RICE MEIOSIS*)^5^, *PAIR2* ^6^, *OsRad21-4* ^7^ and *MEL1* (*MEIOSIS ARRESTED AT LEPTOTENE1*)^8^. Along with meiocytes, anther primordial cells differentiate into four cell layers, epidermis, endothecium, middle layer and tapetum surrounding each lobe. The rice tapetum has a secretory function, the degradation of the tapetum after meiosis provides multiple nutrients for microspore development. Defects in tapetum formation, premature or delayed degradation can result in male sterility. Transcription factors seem to play vital roles in tapetum formation and degradation, such as *Utd1* (*UNDEVELOPED TAPETUM1*)^9^, *TDR* (*TAPETUM DEGENERATION RETARDATION*)^10^ and *MYB*s^11–13^. With the maturation of pollen grains, anther locules are broken at the stomium to release pollen grains. Secondary wall thickenings in the anther endothecium are necessary for anther dehiscence; the special U-shaped thickenings generate tensile forces required for stomium rupture^14^. The MYB transcription factor MYB26^15^ and two NAC transcription factors NST1 (NAC SECONDARY WALL-PROMOTING FACTOR1) and NST2^16^, play important roles in anther dehiscence through regulating secondary wall thickenings in the endothecium. In addition to secondary wall thickenings, suitable humidity and the swelling of pollen grains are required for successful rice anther dehiscence^17^.

Phytochromes are a class of photoreceptors that mainly perceive and respond to red and far-red light to regulate multiple aspects of plant growth and development. Three genes encode phytochromes in the rice genome: phytochrome A (*PHYA*), *PHYB* and *PHYC* ^18–22^. Rice phytochromes are the key regulators that control a series of events during photomorphogenesis, including de-etiolation, plant shape formation, and flowering time^22, 23^. In our previous studies, the *phyA phyB phyC* triple mutant was found to have much lower fertility than the wide-type (WT), whereas the single mutants showed normal fertility^23, 24^. The reduced fertility of the triplet mutant was believed to be caused by the failure of anther dehiscence^23, 24^. In this study, we firstly reported that the mutation of two phytochrome genes, *PHYA* and *PHYB*, could lead to partial male sterile, which was similar to that of the triple mutant. To uncover the regulatory network of *PHYA* and *PHYB* in anther development, a series of histological and cytological observations were carried out, and the results showed that the *phyA phyB* mutant had defects in both anther and pollen development. Subsequently, a comprehensive transcriptome analysis was performed in the present study to compare gene expression profiles between *phyA* and *phyB* single and double mutant, and WT rice anthers. The results revealed that carbohydrate metabolism, stress-, and photosynthesis-related genes were significantly affected in the *phyA phyB* double mutant, indicating a complex regulatory network underlying phytochrome-mediated anther and pollen development in rice.

## Results

### The *phyA phyB* double mutant is defective in anther development

**Figure 1:**
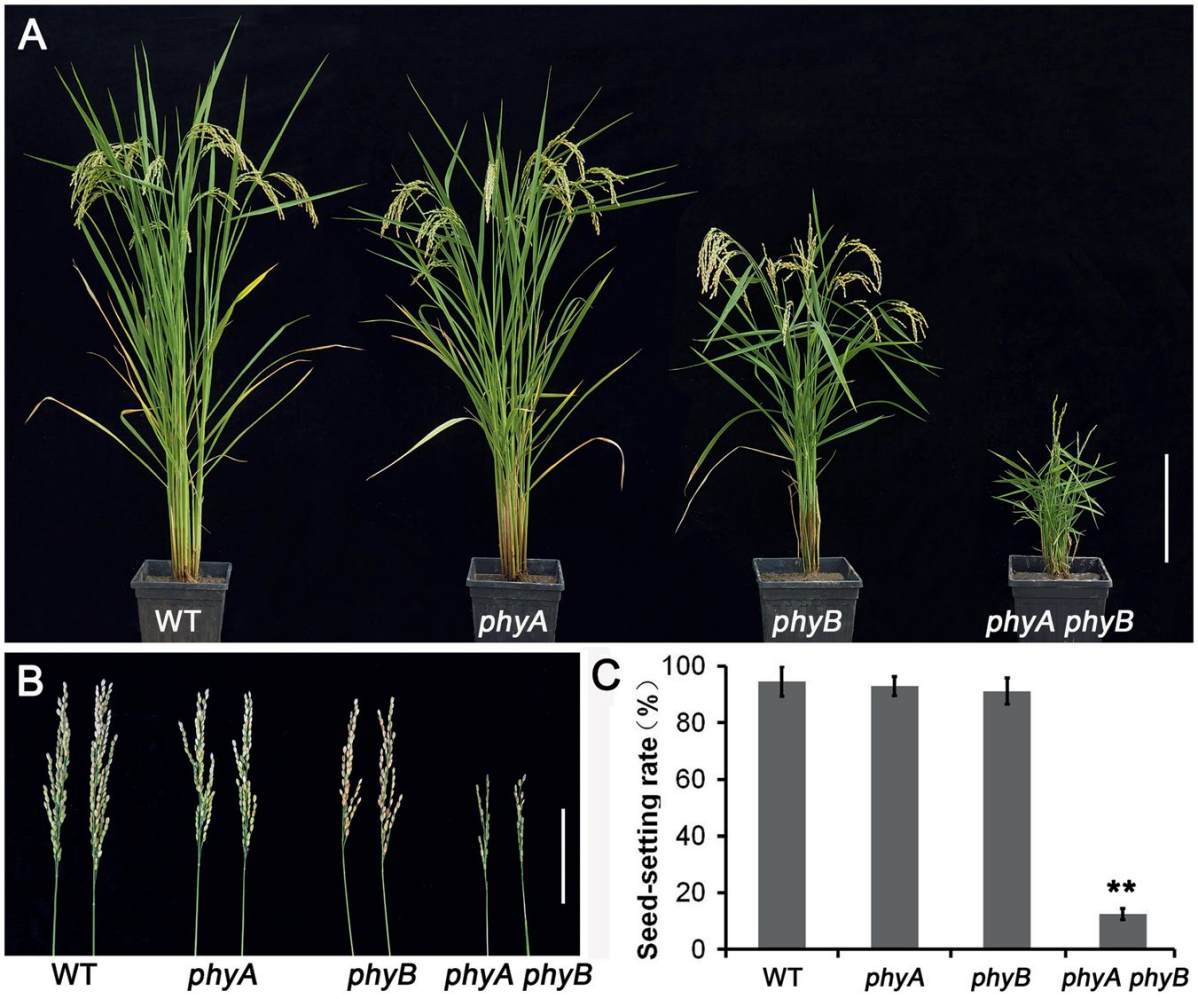
Agricultural traits of the *phyA* and *phyB* single mutants, *phyA phyB* double mutant, and wild-type (WT) rice. (**A**) Comparison of the *phyA* and *phyB* single and double mutants with the WT rice at the grain-filling stage. (**B**) The panicles of *phyA* and *phyB* single and double mutants, and WT rice. (**C**) Statistical analysis of the seed-setting rates of *phyA* and *phyB* single and double mutants, and WT rice. Data were analyzed using paired *t* tests, and **means p value < 0.01. Bars = 20 cm in A, 10 cm in B.

As described in our previous studies, the *phyA phyB phyC* triple mutant showed lower fertility than those of single mutants and WT rice^23, 24^. In this study, we found that the double mutant *phyA phyB*, not *phyA phyC* or *phyB phyC*, was also deficient in seed production. The results indicated that *PHYC* functions redundantly with *PHYA* and *PHYB* in fertility control. In additon, the *phyA phyB* mutant showed significantly different plant architecture-associated characteristics, such as decreased plant height, smaller angle between leaf blade and leaf sheath, smaller and straighter panicles, from those of single mutants and WT (Fig. 1A,B). The seed-setting rate of the *phyA phyB* mutant was significantly lower, approximately 12%, compared with the seed-setting rates of the single mutants and the WT plants, which were over 90% (Fig. 1C).

**Figure 2:**
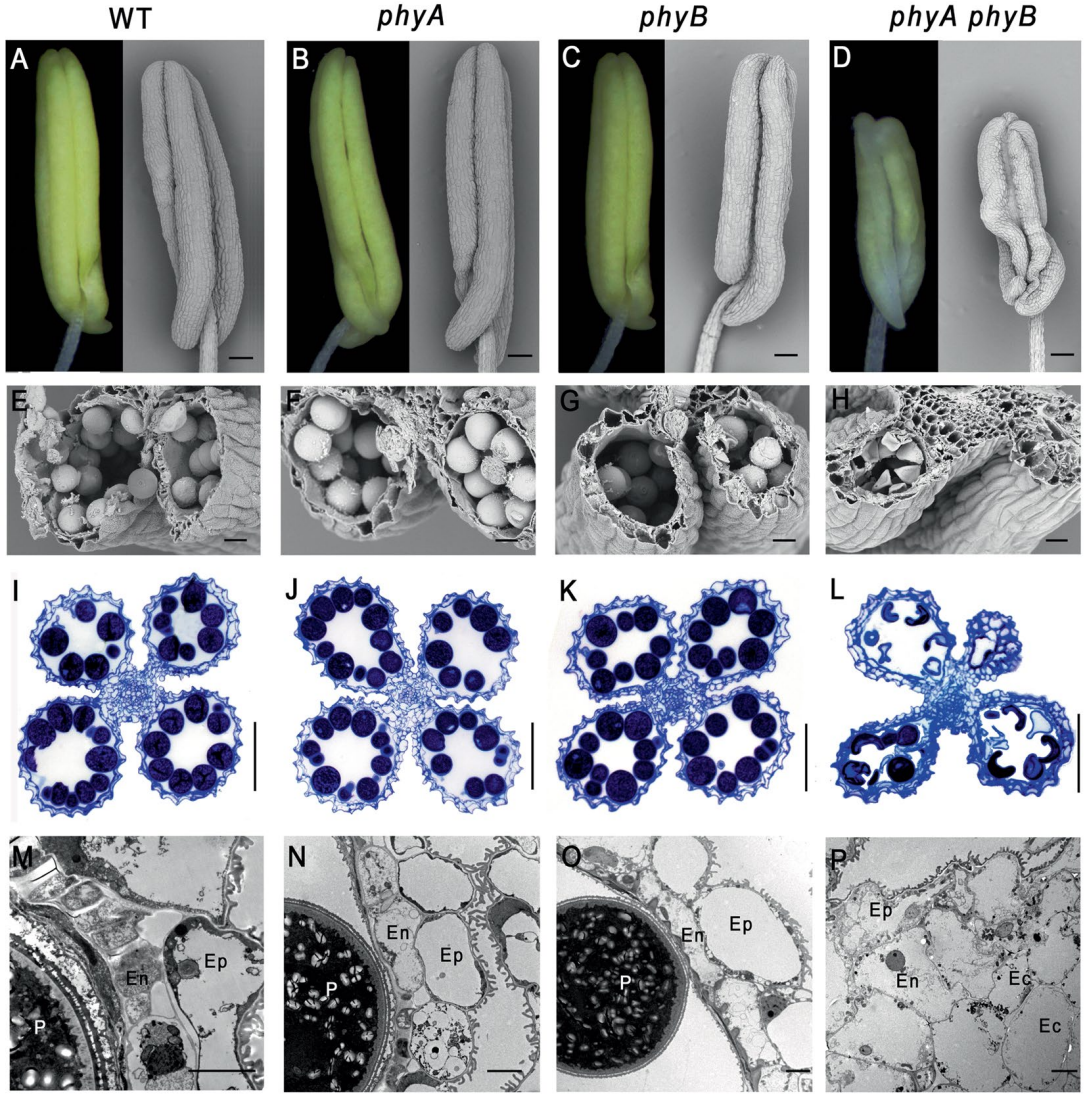
Anthers of *phyA* and *phyB* single and double mutants, and wild-type (WT) rice at stage 12. (**A**–**H**) Light microscopic and scanning electron microscopic analysis of anthers at stage 12. (**I**–**L**) Sections of anthers at stage 12. (**M**–**P**) Transmission electron microscopic analysis of anthers at stage 12. Ep, epidermal cell layer; En, endothecium cell layer; Ec, extra cell layer; P, pollen grain. Bars = 0.1 mm in (**A**–**D**) 20 µm in (**E**–**H**) 50 µm in I–L, 5 µm in M–P.

The reduced fertility of the *phyA phyB* double mutant was attributed to the male reproductive tissues because a reciprocal cross with WT pollen restored the fertility of the mutant. In the present study, detailed histological and cytological observations were performed to analyze the male reproductive tissues of the *phyA phyB* mutant. Compared with the single mutants and WT, anthers of the *phyA phyB* mutant exhibited significant differences, including pale-yellow appearances, smaller sizes (~75% the length of the single mutants and WT), and shriveled and distorted anther lobes (Fig. 2A–D). In addition, both the scanning electron microscopic and the semi-thin section observations of anther structures from the *phyA* and *phyB* single mutants, *phyA phyB* double mutant and WT showed that the structures of the four anther lobes of the double mutant were different from one another (Fig. 2E–L), indicating that their developmental synchronization was interrupted. The four lobes remained at different stages, and one or two lobes even could not produce any visible pollen grains (Fig. 2H,L). Before anthesis, the middle layer and tapetum largely degenerated, leaving only the epidermis, which was covered by a cuticular membrane, and the endothecium, which was deposited by U-shaped ligno-cellulosic thickenings adjacent to two splits in the WT and single mutants (Fig. 2M–O). The deposition of secondary thickenings were also observed in the endothecium of some lobes in the anthers of the *phyA phyB* mutant, but the cell layers of the anther walls were more numerous, at least one lobe was just consisted of several cell layers (Fig. 2H,L and P). The extra cell layers of the anther wall in the *phyA phyB* mutant were consisted of enlarged cells with low cytoplasmic density, which were distinguished from those of the middle layer and tapetum layer cells in single mutants and WT (Fig. 2P).

### Pollen development was severely retarded and pollen viability decreased significantly in the *phyA phyB* mutant

**Figure 3:**
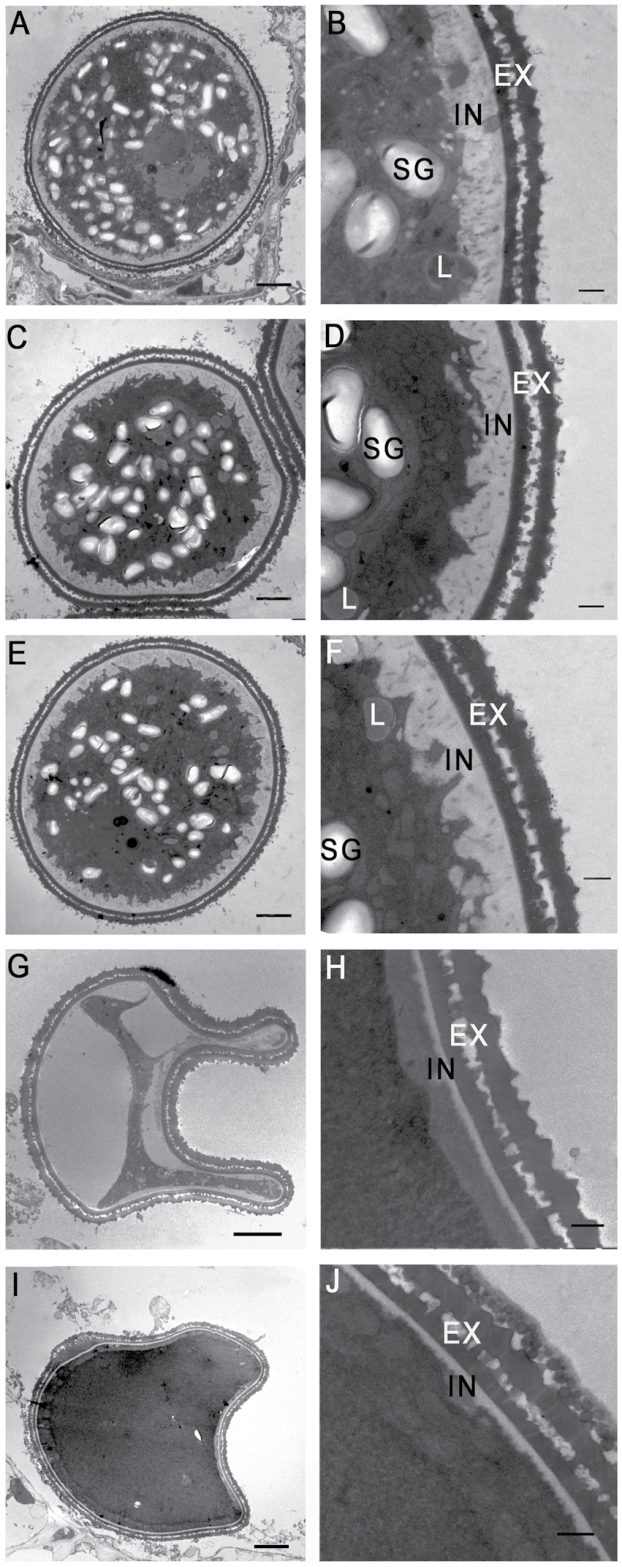
Transmission electron microscopic analysis of pollen grains in the *phyA* and *phyB* single and double mutants, and wild-type (WT) rice at stage 12. Cross sections of pollen grains from WT (**A** and **B**), the *phyA* mutant (**C** and **D**), the *phyB* mutant (**E** and **F**), the *phyA phyB* mutant (**G**–**J**). EX, exine layer; IN, intine layer; SG, starch granule; L, lipid body. Bars = 5 µm in (**A**,**C**,**E**,**G** and **I**) 1 µm in (**B**,**D**,**F**,**H** and **J**).

As shown in Fig. 2H,L, pollen development was affected severely in the *phyA phyB* mutant, resulting in multiple shriveled pollen grains, most of which were arrested at stage 11. To observe pollen defects in the *phyA phyB* mutant more clearly, we used transmission electron microscopy to compare pollen grains among WT, single and double mutants. At the mature stage, pollen grains in WT and single mutants were round and enriched with storage materials, such as starch granules and lipid bodies (Fig. 3A–F), which are necessary for pollen viability and function. However, in the *phyA phyB* mutant, most of the pollen grains were irregular in shape and filled with materials of high or low electronic density, no starch granule were visible (Fig. 3G–J). In addition, the transmission electron micrographs of pollen wall structures of the single mutants, double mutant and WT showed no significant difference in the exine structures. However, the double mutant formed a much thinner intine than those of WT and the single mutants (Fig. 3H,J). Considering that pectin and cellulose are the major constituents of intine, we hypothesize that *phyA phyB* mutant pollens contain much less pectin and cellulose.

**Figure 4:**
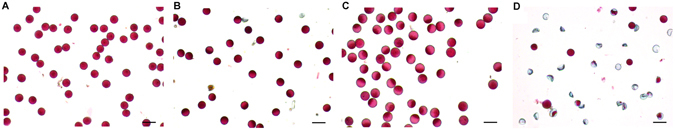
Alexander’s staining of anthers at stage 12. Pollen grains in wild-type (WT) (**A**) the *phyA* mutant (**B**) and the *phyB* mutant (**C**) are stained purple, indicating they are well developed. Most of the pollen grains in the *phyA phyB* mutant are stained green (**D**), indicating they were aborted. Bars = 50 µm.

To examine the vitality of pollen grains in the *phyA phyB* mutant, Alexander’s staining was used to distinguish the viable and aborted pollen grains. As shown in Fig. 4A–C, almost all of the pollen grains in the WT, and *phyA* and *phyB* single mutants were stained purple, indicating that they were active. In contrast, approximately 80% pollen grains in the *phyA phyB* mutant were stained green, which suggested that a large number of pollen grains were aborted (Fig. 4D).

Thus, the reduced fertility of the *phyA phyB* mutant was determined by two factors, one is the abnormal anther development and failure of anther dehiscence, the other one is reduced pollen vitality.

### Overview of anther transcription profiles of *phyA* and *phyB* single and double mutants and WT rice

To identify genes responsible for abnormal anther development in the *phyA phyB* mutant, 12 cDNA libraries used for digital gene expression analysis were constructed using anthers of *phyA* and *phyB* single and double mutants and WT at stage 12. Three biological replicates were established for each material. Over 130 million raw reads were generated from the 12 libraries, with *phyA* mutant libraries producing the fewest raw reads and *phyA phyB* mutant libraries producing the most (Table 1). After removing low quality reads, as well as reads with adaptors or more than one N base, the number of clean reads varied greatly among different libraries, from 8,326,096 in the *phyA* library to 14,102,775 in the WT library (Table 1). The clean reads were then mapped to the rice genome, with no more than one mismatch allowed in the alignment. In total, over 36,000 genes were found to be expressed in rice anther (Table 1). The number of genes expressed in *phyA* and *phyB* single and double mutant anthers was similar to that expressed in WT anthers.

**Table 1:** Overview of reads from *phyA* and *phyB* single and double mutants and wild-type (WT) rice anthers.

Sample	Total reads	clean reads	Unique reads	mapped reads	mapped gene
WT-1	9,436,855	9,390,090	4,424,561	3,561,170	35,537
WT-2	14,145,506	14,102,775	6,092,457	4,661,819	36,744
WT-3	11,040,852	11,007,073	4,980,849	3,874,290	36,331
*phyA*-1	8,349,302	8,326,096	3,842,549	3,110,153	35,049
*phyA*-2	10,689,623	10,657,794	4,887,689	3,761,325	36,172
*phyA*-3	9,497,606	9,455,138	4,378,828	3,422,501	35,814
*phyB*-1	9,988,189	9,954,268	4,502,754	3,684,445	35,858
*phyB*-2	11,908,049	11,888,987	5,127,402	4,073,848	36,650
*phyB*-3	10,511,289	10,471,785	4,986,626	3,893,110	36,314
*phyA phyB*-1	10,601,576	10,563,578	5,088,752	4,111,884	36,738
*phyA phyB*-2	12,680,074	12,655,212	6,053,928	4,771,027	37,696
*phyA phyB*-3	11,793,432	11,765,239	5,751,528	4,490,906	37,620

### Identification of candidate genes responsible for abnormal anther and pollen development in the *phyA phyB* double mutant

**Figure 5:**
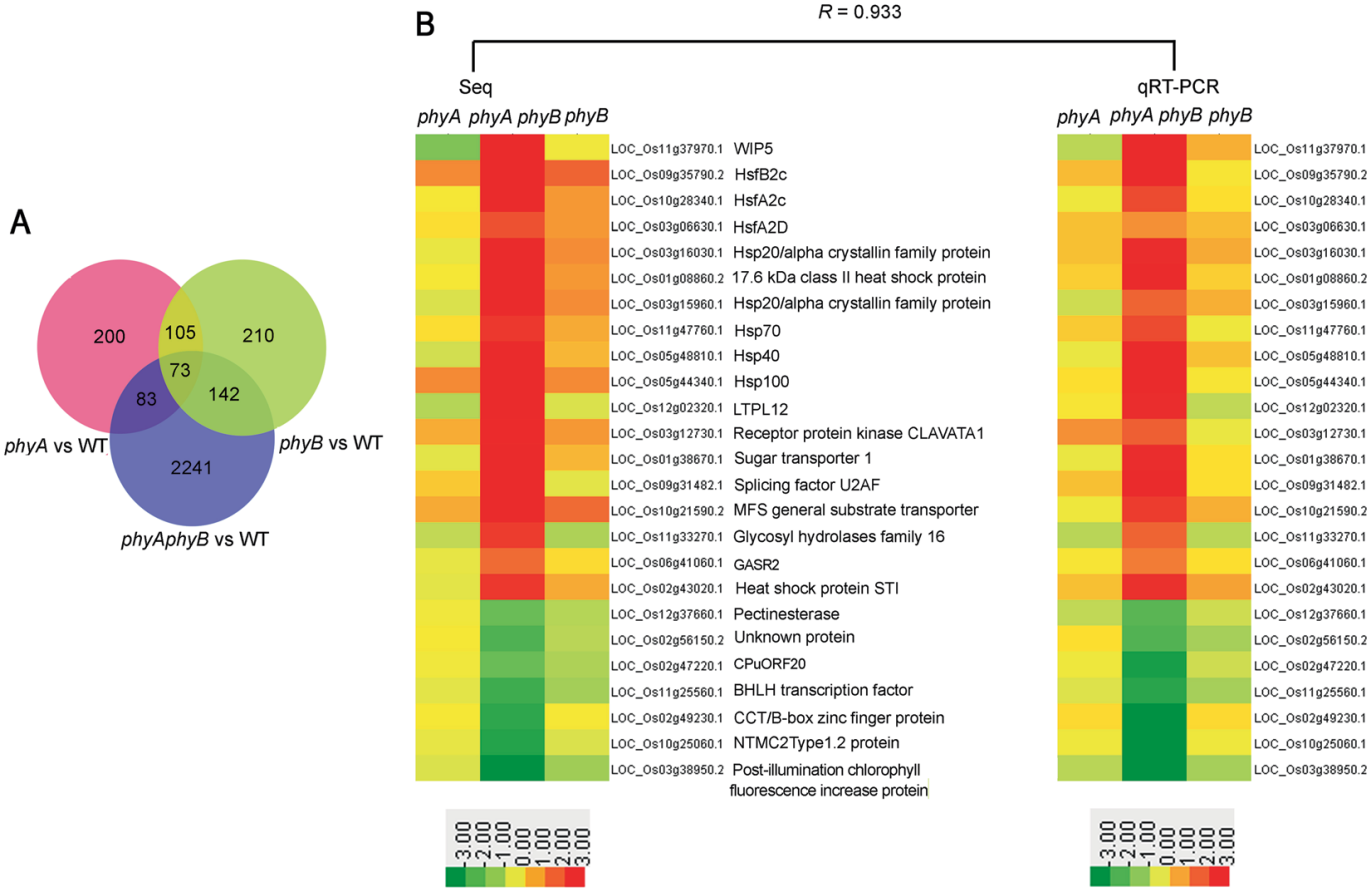
Comparison and validation of transcript profiles of anthers in *phyA* and *phyB* single and double mutants, and wild-type (WT) rice. (**A**) Distribution of differentially expressed genes in anthers among *phyA* and *phyB* single and double mutants, and WT. (**B**) Validation of RNA sequencing results by quantitative reverse transcription PCR. Transcript abundances represented by the two heat maps are the log2-transformed averages of RPKM values of the RNA sequencing analysis (left) from three biological replicates and transcript abundance values from three independent quantitative reverse transcription PCR experiments (right). The heat maps were drawn using Cluster 3.0. Relative expressions are indicated by the color scales at the bottom of the figure. *R* is the correlation coefficient value between the two platforms.

Compared with WT anthers, significantly more genes were differentially expressed in the *phyA phyB* double mutant than in either single mutant. In total, 461 (374 genes), 530 (423 genes), and 2,539 (1,951 genes) differentially expressed transcripts were identified in *phyA*, *phyB* and *phyA phyB* mutants, respectively (Fig. 5A, Table S1). These results indicate that gene expression profiles changes dramatically in the double mutant. Among the 2,539 differentially expressed transcripts between *phyA phyB* double mutant and WT libraries, a small percentage of differentially expressed transcripts (298, 11.74%) were common to *phyA*/WT or *phyB*/WT datasets. The remaining 2,241 differentially expressed transcripts (1,717 genes) were specific to the *phyA phyB*/WT dataset (Fig. 5A). Because a single mutation of *PHYA* or *PHYB* gene has no impact on the fertility, the 2,241 differentially expressed transcripts specific to the *phyA phyB*/WT dataset were considered to be the candidate genes responsible for the abnormal anther and pollen development of the *phyA phyB* double mutant. These *phyA phyB*/WT-specific differentially expressed transcripts were used in subsequent analyses.

Among the candidate transcripts responsible for fertility control in the *phyA phyB* mutant, 1,111 transcripts (847 genes) were up-regulated and 1,130 transcripts (870 genes) were down-regulated (Table S2). Gene Ontology (GO) analysis revealed that genes involved in stimulus responses, carbohydrate metabolism and transport were over-represented in the up-regulated transcripts, while genes involved in photosynthesis were enriched in the down-regulated ones (Fig. S1). Half of genes involved in stimulus responses in the up-regulated dataset encode heat-shock factors (Hsfs) and proteins (Hsps) (Table S2). Among genes involved in carbohydrate metabolism in the up-regulated dataset, genes participate in cellulose degradation take the largest proportion, followed by genes involved in starch metabolism, chitin hydrolysis, galactose catabolism and callose hydrolysis (Table S2).

To confirm gene expression patterns detected by RNA sequencing, 25 genes differentially expressed between the *phyA phyB* mutant and the WT were examined by quantitative reverse transcription-PCR (qRT-PCR). As shown in Fig. 5B, most gene expression patterns detected by qRT-PCR were similar to those revealed by RNA-Seq. Correlation coefficient between the two methods reached to 0.933. The qRT-PCR results indicate that the RNA-Seq results were quite reliable.

### The metabolic profile changed significantly in the *phyA phyB* mutant

**Figure 6:**
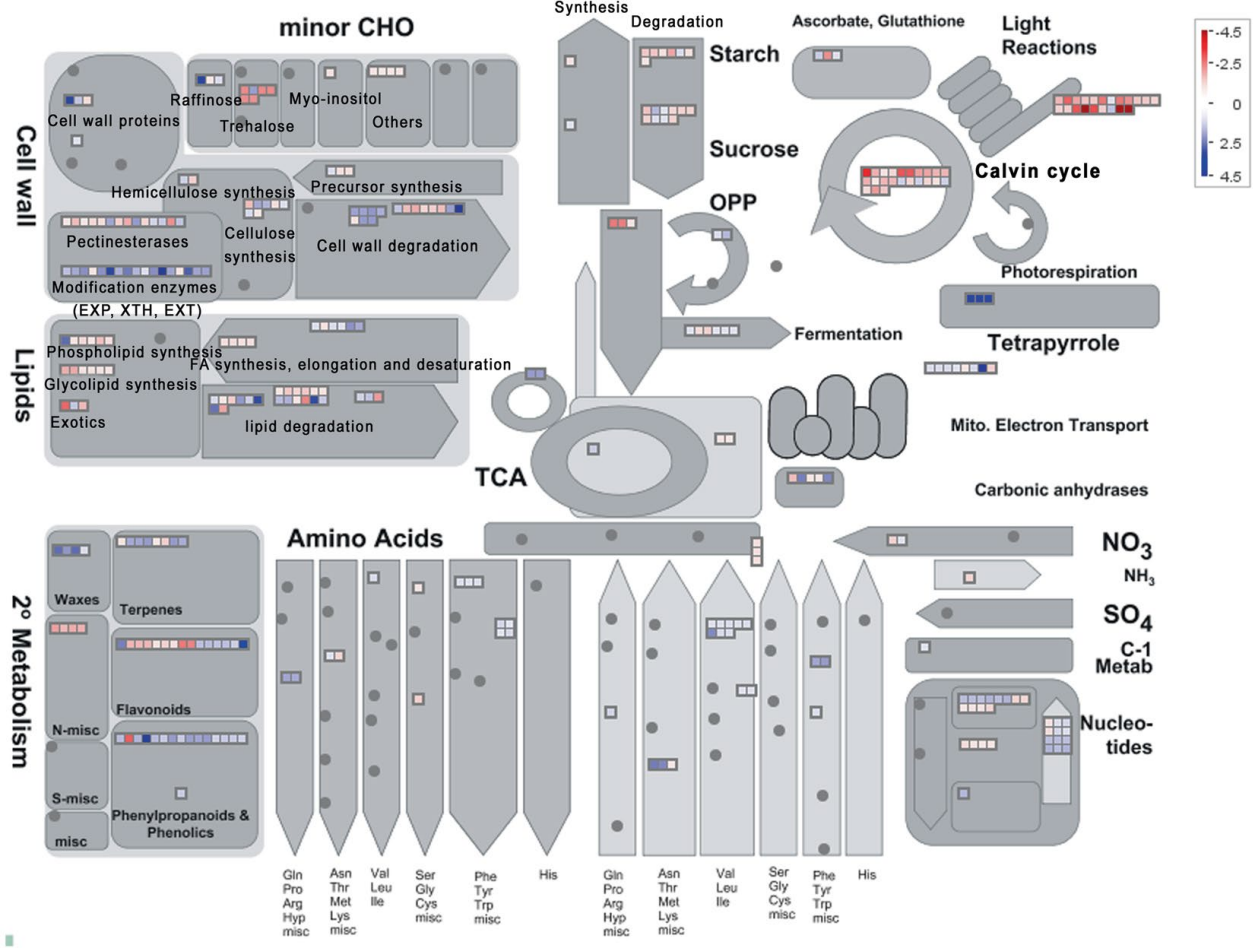
Overview of the metabolic profiles of the differentially expressed transcripts in the *phyA phyB* mutant by MapMan. Transcripts showing different fold-changes between the *phyA phyB* mutant and wild-type (WT) rice were mapped in different colors. Red boxes indicate that transcripts were down-regulated in the *phyA phyB* mutant, while blue boxes indicate that transcripts were up-regulated in the double mutant.

GO analysis of the differentially expressed transcripts that were specific to the *phyA phyB* double mutant revealed that genes involved in metabolic processes, including carbohydrate metabolism and photosynthesis, were over-represented in the *phyA phyB* mutant (Fig. S1). To overview the metabolic profile changes in the *phyA phyB* mutant, we carried out the MapMan analysis tool. As shown in Fig. 6, both major and minor CHO metabolisms were significantly affected in the *phyA phyB* mutant. Starch and sucrose metabolism- and trehalose biosynthesis-related transcripts were mainly down-regulated, which indicated that these sugars changed significantly in the *phyA phyB* mutant. Based on the list of genes up-regulated in the *phyA phyB* mutant, it was found that a large proportion of these genes (35/68, 51.5%) were encoding glycoside hydrolases and β-galactosidases. Members of eight glycoside hydrolase families, 3, 9, 10, 16, 17, 18, 31 and 38, were up-regulated in the *phyA phyB* double mutant. The up-regulated β-galactosidases belonged to families 11, 17 and 44. Considering that enzymes of glycoside hydrolase families 3, 9, 10 and β-galactosidases can hydrolyze cellulose, cellulose hydrolysis should be accelerated in the *phyA phyB* mutant. Two genes encoding cellulase, which mainly contributes to cellulose decomposition in plants, were also up-regulated. This result was in accordance with the abnormal anther walls and the thinner intines of *phyA phyB* mutant pollen grains. In addition, genes involved in two steps of photosynthesis, light reactions and the calvin cycle, were down-regulated significantly (Fig. 6), indicating the *phyA phyB* mutant produced much less organic materials such as sugars, lipids and proteins, than WT. Most of the differentially expressed genes that participated in fatty acid synthesis, elongation and desaturation, and phospholipid and glycolipid synthesis were down-regulated (Fig. 6). In accordance with this result, the *phyA phyB* mutant accumulated lower levels of storage materials in the mature pollen grains (Fig. 3H). In contrast, most of the differentially expressed transcripts related to nucleotide metabolism, amino acid metabolism, wax, terpene and flavonoid metabolisms, were significantly up-regulated (Fig. 6). The expression patterns of cell wall-related transcripts were also changed significantly in the *phyA phyB* mutant. Cell wall modification enzymes, including expansin, xyloglucan endotransglycosylase and endoxyloglucan transferase, were up-regulated, while most of the genes encoding pectinesterase were down-regulated (Fig. 6). All these four enzymes have cell wall remodeling activities, therefore, changes in the expression levels of genes encoding these enzymes may lead to altered anther and pollen wall structures and components in the *phyA phyB* mutant.

### Hsps and Hsfs may play import roles in the anther and pollen development in the *phyA phyB* mutant

Hsps are a class of universal molecular chaperons that play important roles in various biological activities, such as protein folding, assembly and translocation, and degradation of truncated and misfolded proteins. Hsps can also protect plants against various abiotic stresses by assisting plants to reestablish cellular homeostasis^25–27^. Hsps from four major families have been identified in rice, including 29 small Hsp (sHsp), 26 Hsp70, 9 Hsp90 and 10 Hsp100 proteins^28^.

According to the list of up-regulated genes in the *phyA phyB* double mutant, Hsps formed the largest proportion of genes involved in abiotic stress (Table S2). As shown in Table 2, 38 transcripts (25 genes) were Hsps related to abiotic stress responses. Four categories of Hsps were identified in the up-regulated gene set, including 8 sHsp, 5 Hsp40, 5 Hsp70, 6 Hsp90 and 1 Hsp100 (Table 2). In contrast, fewer Hsps were down-regulated in the *phyA phyB* double mutant, except for four genes encoding Hsp40 (Table S2). To verify the Hsp expression patterns in rice anthers, qRT-PCR was adopted. As shown in Fig. 5B, all six Hsps were up-regulated significantly in the *phyA phyB* double mutant, which was consistent with the RNA-Seq results.

**Table 2:** Heat-shock proteins and heat-shock factors up-regulated in the *phyA phyB* mutant.

Gene ID	Annotation	Mean WT RPKM	Mean *phyA* RPKM	Mean *phyB* RPKM	Mean *phyA phyB* RPKM	log2 fold_change	pvalue	FDR
LOC_Os03g15960.1	sHsp	1.58	1.476667	3.846667	20.78	3.38	0	0
LOC_Os03g15960.2	sHsp	1.43	1.32	3.393333	18.45	3.38	0	0
LOC_Os03g16030.1	sHsp	0.97	1.05	2.366667	10.43	3.14	0	0
LOC_Os06g14240.1	sHsp	0.04	0.263333	0.083333	2.51	5.47	0	0
LOC_Os03g16020.1	sHsp	1.05	1.33	3.166667	13.84	3.29	0	0
LOC_Os06g11610.1	sHsp	0.32	0.456667	0.156667	2.11	2.49	0	0.01
LOC_Os03g16040.1	sHsp	1.23	0.88	2.91	9.87	2.73	0	0
LOC_Os02g52150.1	sHsp	0.42	0.086667	1.543333	3.69	2.86	0	0
LOC_Os02g52150.2	sHsp	0.42	0.086667	1.606667	3.94	2.95	0	0
LOC_Os01g08860.1	sHsp	2.39	2.993333	5.37	26.62	3.16	0	0
LOC_Os01g08860.2	sHsp	2.42	2.973333	5.233333	26.91	3.15	0	0
LOC_Os02g54130.2	hsp40	57.31	21.86	3.77	1.45	1.05	0	0.01
LOC_Os03g57340.1	hsp40	57.67	61.49	75.74	160.05	1.15	0	0
LOC_Os06g09560.1	hsp40	4.04	3.143333	6.273333	38.96	2.95	0	0
LOC_Os03g18200.1	hsp40	2.24	2.883333	2.623333	6.62	1.24	0	0
LOC_Os03g18200.2	hsp40	4.95	6.266667	5.466667	15	1.28	0	0
LOC_Os05g48810.1	hsp40	1.56	1.38	2.67	17.37	3.15	0	0
LOC_Os01g08560.1	hsp70	7.38	8.876667	10.49667	23.53	1.35	0	0
LOC_Os01g08560.2	hsp70	7.32	8.726667	10.33333	23.16	1.34	0	0
LOC_Os03g02260.1	hsp70	7.07	9.496667	13.27333	33.06	1.9	0	0
LOC_Os03g11910.1	hsp70	1.05	1.33	1.38	5.72	2.13	0	0
LOC_Os03g11910.2	hsp70	0.97	1.296667	1.323333	5.45	2.16	0	0
LOC_Os05g23740.1	hsp70	8.16	9.676667	12.37667	28.62	1.49	0	0
LOC_Os11g47760.1	hsp70	10.79	14.03333	20.43333	63.52	2.23	0	0
LOC_Os11g47760.2	hsp70	9.68	12.56667	18.61	56.57	2.22	0	0
LOC_Os11g47760.3	hsp70	10.34	13.39667	19.36	60.43	2.22	0	0
LOC_Os11g47760.4	hsp70	11.25	14.66333	21.09333	66	2.23	0	0
LOC_Os11g47760.5	hsp70	10.28	13.35	19.17	59.89	2.22	0	0
LOC_Os11g47760.6	hsp70	6.62	8.57	12.74333	40.32	2.29	0	0
LOC_Os08g39140.1	hsp90	6.89	8.313333	14.36	41.67	2.27	0	0
LOC_Os08g39140.3	hsp90	4.83	5.813333	10.08667	29.28	2.28	0	0
LOC_Os09g29840.1	hsp90	3.03	3.436667	4.566667	9.14	1.26	0	0
LOC_Os09g29840.2	hsp90	2.92	3.253333	4.296667	8.76	1.26	0	0
LOC_Os09g30418.1	hsp90	57.51	51.02	86.66333	182.73	1.35	0	0
LOC_Os09g36420.1	hsp90	1.82	1.8	2.953333	6.01	1.39	0	0
LOC_Os12g32986.1	hsp90	9.83	12.34667	16.92	37.79	1.62	0	0
LOC_Os09g30412.1	hsp90	54.24	48.45333	83.30333	171.33	1.34	0	0
LOC_Os05g44340.1	hsp100	0.18	0.453333	0.43	8.17	5.21	0	0
LOC_Os10g28340.1	HsfA2c	0.33	0.393333	0.743333	2.66	2.69	0	0
LOC_Os10g28340.2	HsfA2c	0.57	0.526667	1.026667	4.04	2.5	0	0
LOC_Os10g28340.3	HsfA2c	0.58	0.54	0.85	3.84	2.41	0	0
LOC_Os10g28340.5	HsfA2c	0.58	0.56	0.89	3.94	2.39	0	0
LOC_Os03g06630.1	HsfA2d	0.76	1.03	1.636667	3.5	1.89	0	0
LOC_Os03g06630.2	HsfA2d	0.8	1.073333	1.66	3.54	1.82	0	0
LOC_Os08g43334.1	HsfB2b	0.31	0.21	0.43	2.24	2.53	0	0
LOC_Os08g43334.2	HsfB2b	0.49	0.353333	1.093333	4.65	2.92	0	0
LOC_Os09g35790.1	HsfB2c	0.06	0.233333	0.336667	4.34	6.02	0	0
LOC_Os09g35790.2	HsfB2c	0.13	0.34	0.486667	5.8	5.17	0	0
LOC_Os09g35790.3	HsfB2c	0.04	0.103333	0.183333	2.5	5.96	0	0
LOC_Os09g35760.2	HsfB2c	0.39	0.5067	0.733	1.24	1.29	0	0.01

As we know, the expression of *Hsp*s is activated by Hsfs which interact with heat shock elements in the promoter regions of Hsps^29^. Consistent with the up-regulation of Hsps in the *phyA phyB* mutant, five *Hsf*s, namely *OsHsfA2c*, *OsHsfA2d*, *OsHsfB2b* and *OsHsfB2c*, were also up-regulated significantly in the double mutant (Table 2). In addition, only one Hsf, *OsHsfC2b*, was down-regulated in the *phyA phyB* double mutant (Table S2). The qRT-PCR results confirmed the expression of the three *Hsf*s (Fig. 5B). The up-regulation of both *Hsf*s and *Hsp*s indicate that they might be the key regulators of anther development in the *phyA phyB* mutant.

## Discussion

Phytochromes are a class of photoreceptors responsible for perceiving red/far-red light in plants. In rice, the mutations of phytochromes not only lead to abnormal photomorphogenesis, but also result in reproductive defects, such as small panicles, infrequent anther dehiscence and low seed-setting^23, 24^. Previous studies have demonstrated that failure of fertilization in the *phyA phyB phyC* mutant was caused by impaired dehiscence of the anther wall^23, 24^. This study firstly reported that the *phyA phyB* double mutant also had defect in male fertility, which is similar to that of triple mutant. To identify genes involved in the phytochrome-mediated reproduction process, a series of cytological observations and RNA-Seq investigations of *phyA* and *phyB* single and double mutants, and WT were carried out in this study. Cytological examination of slides of developing anthers and Alexander’s staining of pollen grains revealed that *phyA* and *phyB* single mutants showed similar anther structures and pollen viability as those of WT (Figs 2, 3 and 4). However, the *phyA phyB* double mutant exhibited hysteretic anther differentiation, more cell layers in at least one lobe, and fewer pollen grains—most arrested at stage 11 in the relatively normal lobes (Fig. 2H,L). These results suggest the existence of novel signaling pathways regulating anther development through phytochromes.

PIFs are a class of basic helix-loop-helix transcription factors that bind directly to phytochromes in the biologically active Pfr form^30^. They usually localize to the nucleus and bind specifically to downstream genes at the light-regulated cis-element G-box and PBE-box in their promoter regions^31–35^. Thus, identifying PIFs and their downstream genes under the regulation of phytochromes is an efficient way to establish novel phytochrome-mediated signaling pathways. In this study, two PIFs were specifically differentially expressed in the *phyA phyB* mutant (Table S2), which indicates they function downstream of *PHYA* and *PHYB* in rice. The two PIFs showed opposite expression patterns; *PIF16* was up-regulated significantly in the *phyA phyB* mutant, whereas *PIF14* were down-regulated (Table S2). To identify the candidate genes targeted by the two PIFs, we analyzed the distribution of putative PIF-binding motifs (including G-box and PBE-box) in the 2 kb upstream regions of the 2,241 differentially expressed transcripts (1,717 genes) specific to the *phyA phyB* double mutant. The result showed that more than half of the differentially expressed genes had at least one G-box or PBE-box in their promoter regions (Fig. S2). In detail, 566 (32.7%) and 912 (53.1%) differentially expressed genes had at least one G-box and PBE-box in their promoter regions, respectively. Among them, 319 genes had both the two *cis*-elements in their promoter regions. The large number of differentially expressed genes with G-box and/or PBE-box in their promoter regions might act as direct targets of the two PIFs in the signaling pathways of *PHYA-* and *PHYB-*mediated rice growth and development.

Environmental stresses, such as heat, drought and cold, have adverse impacts on anther development, especially during meiosis and mitosis^36–39^. Wheat exposed to elevated temperatures during pollen mitosis 1 and 2 triggers two types of abnormal anther development^40^. In one type, tapetum degradation occurs during meiosis, but the first microspore mitosis could not finish, resulting in immature pollen grains with normal exine but no cytoplasm. In the other type, the first mitotic division of the microspores proceeds to completion; however, few microspores undergo complete second mitosis, with mostly sterile pollen grains as a consequence^41^. Prolonged cold treatment of rice at the young microspore stage leads to small anthers and severe male sterility, which results from the hypertrophy and break down of tapetum cells^42^. In major cereals, such as wheat, maize, and rice, water deficit at the meiotic stage leads to partial or complete male sterility through inhibition of the future development of microspores and pollen grains^37, 43, 44^. The failure of male gametophyte development under stress treatments was considered to be the result of impaired carbohydrate metabolism^43–45^.

Carbohydrate metabolism disturbance severely affects anther development, even lead to male sterility^46, 47^. Sugars are the key components of developing anthers, especially in the anther wall fraction where sugar synthesis, secretion, storage, mobilization, and regulation take place^48^. At the early stage of rice anther development, starch is the most abundant sugar in the endothecium; it then decreases after meiosis, but reaches a much higher level during the first pollen grain mitosis^44^. In addition, cellulose and lignified thickenings, which are essential for anther dehiscence, are deposited in the endothecium during microspore maturation^49^. During rice anther maturation, reducing sugar contents increase while those of non-reducing sugars decrease^44^. Water deficit has no obvious effect on starch content at the meiotic stage, but tends to have a significant inhibitory effect during later stages. After first mitosis, starch content in drought-stress-treated anthers is less than half that of control plants^44^. Following meiosis, reducing sugar contents of drought-stress-treated anthers during subsequent stages are lower than controls, whereas non-reducing sugar contents are higher^44^. Comparing the expression profiles between *phyA*, *phyB* and *phyA phyB* mutants and that of WT revealed that the carbohydrate metabolic profiles changed significantly in the double mutant. Major and minor CHO metabolisms were altered greatly, and photosynthesis was inhibited in the *phyA phyB* mutant (Fig. 6). Examinations of anthers and pollens in the *phyA phyB* double mutant showed similar results. In the *phyA phyB* mutant, more than one anther lobe did not generate tapetum which supplies nutrients, such as sugars, lipids and proteins, for pollen development and maturation^47^. Instead, these anther lobes produced several cell layers in anther locules without any visible pollen grains (Fig. 2H,L). In addition, the *phyA phyB* mutant produced much less pollen grains, most of which were arrested at stage 11 (Fig. 2H,L). Transmission electronic microscopy analyses revealed that the pollen intines were much thinner, and the storage materials, especially starches and lipids, were absent in the *phyA phyB* mutant (Fig. 3G–J). The cytological observations of the anthers and pollen grains of the double mutant indicated its metabolism changed significantly, with sharp decreases in cellulose, starch and lipid contents.

According to our study, the mutations of *PHYA* and *PHYB* seem to have impacts on anther development similar to those of stress treatments. In this study, 28 *Hsp*s and 5 *Hsfs* were up-regulated in the *phyA phyB* mutant (Table 2), which indicated that *PHYA* and *PHYB* mutations altered rice sensitivities to environmental stress. Consistent with this hypothesis, *phyBD*, *phyABDE* and *phyABCD* showed sequentially enhanced resistance to abscisic acid and NaCl treatments in *Arabidopsis* ^50^. Similarly, the *phyA phyB* mutant in rice exhibited less sensitivity to NaCl and low temperature stresses than WT and single mutants (Unpublished data). Previous studies revealed that Hsps and Hsfs play important roles in anther and pollen development. Many Hsps are expressed at different stages of anther development in various plant species, such as *Arabidopsis*, tobacco and tomato^51–54^. For example, the Hsp40 protein TMS1 is required for normal pollen tube growth under heat shock environments in *Arabidopsis* ^55^. In tobacco, two small Hsps are up-regulated significantly in the transition from unicellular to bicellular pollen, while nine other small Hsps are down-regulated at the same stage in tobacco^54^. *HsfA2* and *Hsp17-CII* are activated at the tomato diploid pollen mother cell stage, and their expression levels are up-regulated significantly under short and prolonged heat shock treatments in subsequent anther developmental stages^56^. Considering both the abiotic stress treatments and the absence of phytochromes could rapidly induce the expression levels of stress-responsive genes, such as *HSF*s and *HSP*s, we suggest that the phytochrome status has a great impact on plant stress responses. Previous studies revealed that male sterility induced by abiotic stress treatments were caused by impaired carbohydrate metabolisms in many plant species^43–45^. In this study, we also found that the carbohydrate metabolism profiles changed significantly in the *phyA phyB* mutant. Thus, we inferred that the growth and development of the double mutant wasted much carbohydrates and energy, and that the male sterility of the double mutant resulted from the imbalanced carbohydrate metabolism.

## Methods

### Plant materials and growth conditions

Four plant materials were used in this study: WT rice (*Oryza sativa* L. cv. Nipponbare), *phyA* (*phyA-4*), *phyB* (*phyB-1*) and *phyA phyB* (*phyA-4 phyB-1*) mutants^21, 22^. The *phyA* mutant was obtained by screening *Tos17* retrotransposon mutant panels^21, 22^, which has a *Tos17* insertion in the middle of the second exon of the *PHYA* gene. The *phyB* mutant was isolated from γ-ray-mutagenized rice seeds^22^, which has 1 bp insertion (C) in the the first exon. All the mutants used in this study were homozygous in the background of Nipponbare. Seeds were surface sterilized in 70% (v/v) ethanol for 30 s and then placed in 5% NaClO (v/v) for 20 min. After rinsing six times in sterile double-distilled water, the seeds were incubated in the dark at 28 °C for 3 days to induce germination. During July to August, seedlings were transplanted into soil at the farm of the Shandong Academy of Agricultural Sciences in Jinan, Shandong, China (latitude 36°40′N; longitude 117°00′E). Anthers from the four plant materials were collected before anthesis. The collected anthers were frozen in liquid nitrogen and transferred to a −80 °C freezer until RNA extraction.

### Anther structural analysis and pollen viability test

Anthers from the WT, *phyA*, *phyB* and *phyA phyB* mutants were fixed before anthesis as described by Li *et al*.^10^. Then they were embedded in Spurr’s resin (Sigma-Aldrich, St Louis, MO). Semi-thin sections were generated with an Ultracut E ultramicrotome (Leica Microsystems). After staining with 0.5% (w/v) toluidine blue-O (Sigma-Aldrich, St Louis, MO), the sections were observed and photographed under an Olympus BX51 microscope.

Transmission electron microscopic analysis of anthers from WT, *phyA* and *phyB* single, and *phyA phyB* double mutants were sampled at stage 12 and fixed for 4 h in phosphate buffer, at pH 7.0, supplemented with 2.5% glutaraldehyde. Then they were rinsed with the same buffer and post-fixed for 2 h in phosphate buffer containing 1% osmium tetroxide (Pelco International, Redding, CA, USA) at room temperature. After dehydration by a graded ethanol series (50%, 70%, 80%, 90%, 95% and 100%), the specimens were embedded in Spurr resin (SPI). Ultrathin sections (50 to 70 nm thick) collected on uncoated nickel grids (100 mesh) were stained with 2% uranyl acetate and 1% lead citrate, then examined at 80 kV using a transmission electron microscope (Hitachi 7650, Ibaraki, Japan).

The samples used for scanning electron microscopic analysis were the same as those of used in transmission electron microscopic analysis. All the samples were embedded for 4 h in 0.1 M sodium phosphate buffer solution containing 2.5% glutaraldehyde, at pH 7.0, rinsed three times in 0.1 M phosphate buffer, at pH 7.0, and further fixed in 0.1 M sodium phosphate buffer solution containing 1% osmium tetroxide for 2 h. They were rinsed again in the same buffer, dehydrated in an acetone series from 50% to 100% and then rinsed three times with 100% acetone, which was exchanged three times with isoamyl acetate (Sigma-Aldrich). They were then processed for critical-point drying using liquid CO_2_, and gold-coated (10-nm thickness) in an E-1045 sputter coater (Hitachi, Tokyo, Japan). The specimens were then examined in a TM3000 (Hitachi, Tokyo, Japan) with an accelerating voltage of 15 kV.

For the pollen viability test, anthers were stained in Alexander stain for 1~2 days according to Alexander (1969)^57^.

### RNA extraction, cDNA library construction, and sequencing

Total RNA was extracted from rice anthers using the modified CTAB method as described in Xu *et al*.^58^. The quantity and purity of total RNA were analyzed on a Bioanalyzer 2100 instrument (Agilent, Santa Clara, CA, USA). RNAs with integrity numbers above 7.0 were used for library construction. Approximately 10 μg total RNA was used for generating cDNA according to the Illumina protocol (Illumina, Aan Diego, CA, USA). The products were single-end sequenced on an Illumina Hiseq. 2500 sequencer at LC Sciences (Hangzhou, China) following the vendor’s recommended protocol. Processing of fluorescent images into sequences, base-calling and quality value calculations were carried out using the Illumina data processing pipeline (version 1.8).

### Read alignment and assembly

Raw reads containing adaptor sequences, with low quality and unknown nucleotides N were filtered to obtain clean reads. The clean reads were then subjected to quality assessment, which included classification of total and distinct reads and percentage counts in libraries, saturation analysis of libraries and correlation of biological replicates. All clean reads were mapped to the rice genome (http://phytozome.jgi.doe.gov/pz/portal.html) using Bowtie (version 1.0.0), with only one base-pair mismatch allowed. The number of perfect clean reads corresponding to each gene was calculated and normalized to the number of RPKMs^25^.

### Differentially expressed genes identification and functional analysis

Transcript profiles of all the sequencing data were analyzed by calculating the number of reads per kilobase of exon model per million mapped reads (RPKM)^59^. To identify candidate genes involved in anther development in the *phyA phyB* mutant, a series of comparisons between *phyA* and *phyB* single and double mutants, and WT anther expression profiles were carried out using DEseq.^60^. Transcripts with fold change ≥2, FDR ≤ 0.01 and read number ≥20 in at least one of the two comparison samples were considered to be differentially expressed. Gene Ontology (GO) analyses were performed using AgriGO^61^.

### qRT-PCR analysis

Three biological replicates of total RNA were used for qRT-PCR analysis. Total RNA was extracted as described above. After DNase I treatment, approximately 4 μg of total RNA was reverse transcribed using M-MLV RTase cDNA syntheis kit (Takara, Dalian, China).

qRT-PCR was performed on a StepOne plus Real-Time PCR system (ABI, Carlsbad, CA, USA) using SYBR Green PCR master mix (Takara, Dalian, China). The *EF-1α* gene was used as an internal control. Primers used in qRT-PCR experiments were listed in Table S3.

## Electronic supplementary material

Supplementary Information

Table S1

Table S2

Table S3

## References

[1] IRGSP (2005). The map-based sequence of the rice genome. Nature.

[2] Zhang DB, Wilson ZA (2009). Stamen specification and anther development in rice. Chinese Sci. Bull..

[3] Sanders PM (1999). Anther developmental defects in *Arabidopsis thaliana* male-sterile mutants. Sex. Plant Reprod..

[4] Scott RJ, Spielman M, Dickinson HG (2004). Stamen structure and function. Plant Cell.

[5] Nonomura K (2004). The novel gene *Homologous Pairing Aberration in Rice Meiosis1* of rice encodes a putative coiled-coil protein required for homologous chromosome pairing in meiosis. Plant Cell.

[6] Nonomura K, Nakano M, Eiguchi M, Suzuki T, Kurata N (2006). PAIR2 is essential for homologous chromosome synapsis in rice meiosis I. J. Cell Sci..

[7] Zhang L, Tao J, Wang S, Chong K, Wang T (2006). The rice OsRad21-4, an orthologue of yeast Rec8 protein, is required for efficient meiosis. Plant Mol. Biol..

[8] Nonomura K (2007). A germ cell specific gene of the ARGONAUTE family is essential for the progression of premeiotic mitosis and meiosis during sporogenesis in rice. Plant Cell.

[9] Jung KH (2005). Rice *Undeveloped Tapetum1* is a major regulator of early tapetum development. Plant Cell.

[10] Li N (2006). The rice *Tapetum Degeneration Retardation* gene is required for tapetum degradation and anther development. Plant Cell.

[11] Kaneko M (2004). Loss-of-function mutations of the rice *GAMYB* gene impair alpha-amylase expression in aleurone and flower development. Plant Cell.

[12] Millar AA, Gubler F (2005). The Arabidopsis *GAMYB-like* genes, *MYB33* and *MYB65*, are microRNA-regulated genes that redundantly facilitate anther development. Plant Cell.

[13] Tsuji H (2006). GAMYB controls different sets of genes and is differentially regulated by microRNA in aleurone cells and anthers. Plant J..

[14] Keijzer CJ (1987). The processes of anther dehiscence and pollen dispersal. I. The opening mechanism of longitudinally dehiscing anthers. New Phytol..

[15] Yang C (2007). Arabidopsis *MYB26/MALE STERILE35* regulates secondary thickening in the endothecium and is essential for anther dehiscence. Plant Cell.

[16] Mitsuda N, Seki M, Shinozaki K, Ohme-Takagi M (2005). The NAC transcription factors NST1 and NST2 of *Arabidopsis* regulate secondary wall thickenings and are required for anther dehiscence. Plant Cell.

[17] Matsui T, Omasa K, Horie T (1999). Mechanism of anther dehiscence in rice (*Oryza sativa* L.). Ann. Bot..

[18] Basu D (2000). Rice PHYC gene: structure, expression, map position and evolution. Plant Mol.Biol..

[19] Dehesh K, Tepperman J, Christensen AH, Quail PH (1991). phyB is evolutionarily conserved and constitutively expressed in rice seedling shoots. Mol. Gen. Genet..

[20] Kay SA, Keith B, Shinozaki K, Chua NH (1989). The sequence of the rice phytochrome gene. Nucleic Acids Res..

[21] Takano M (2001). Isolation and characterization of rice phytochrome A mutants. Plant Cell.

[22] Takano M (2005). Distinct and cooperative functions of phytochromes A, B, and C in the control of deetiolation and flowering in rice. Plant Cell.

[23] Takano M (2009). Phytochromes are the sole photoreceptors for perceiving red/far-red light in rice. Proc. Natl. Acad. Sci. USA.

[24] Gu J, Liu J, Xue Y, Zang X, Xie X (2011). Functions of phytochrome in rice growth and development. Rice Sci..

[25] Boston RS, Viitanen PV, Vierling E (1996). Molecular chaperones and protein folding in plants. Plant Mol.Biol..

[26] Vierling E (1991). The roles of heat shock proteins in plants. Annu. Rev. Plant Biol..

[27] Waters ER, Lee GJ, Vierling E (1996). Evolution, structure and function of the small heat shock proteins in plants. J. Exp. Bot..

[28] Hu W, Hu G, Han B (2009). Genome-wide survey and expression profiling of heat shock proteins and heat shock factors revealed overlapped and stress specific response under abiotic stresses in rice. Plant Sci..

[29] Nover L (2001). Arabidopsis and the heat stress transcription factor world: how many heat stress transcription factors do we need?. Cell Stress Chaperone.

[30] Duek PD, Fankhauser C (2005). bHLH class transcription factors take centre stage in phytochrome signaling. Trends Plant Sci..

[31] Martínez-García JF, Huq E, Quail PH (2000). Direct targeting of light signals to a promoter element-bound transcription factor. Science.

[32] Hornitschek P (2012). Phytochrome interacting factors 4 and 5 control seedling growth in changing light conditions by directly controlling auxin signaling. Plant J..

[33] Oh E, Zhu JY, Wang ZY (2012). Interaction between BZR1 and PIF4 integrates brassinosteroid and environmental responses. Nat. Cell Biol..

[34] Zhang Y (2013). A quartet of PIF bHLH factors provides a transcriptionally centered signaling hub that regulates seedling morphogenesis through differential expression-patterning of shared target genes in *Arabidopsis*. PLoS Genet..

[35] Pfeiffer A, Shi H, Tepperman JM, Zhang Y, Quail PH (2014). Combinatorial Complexity in a Transcriptionally Centered Signaling Hub in Arabidopsis. Mol. Plant.

[36] Boyer JS, Westgate ME (2004). Grain yields with limited water. J. Exp. Bot..

[37] Saini HS, Aspinall D (1981). Effect of water deficit on sporogenesis in wheat (*Triticum aestivum* L.). Ann. Bot..

[38] Saini HS, Westgate ME (1999). Reproductive development in grain crops during drought. Adv. Agron..

[39] Stone, P. The effects of heat stress on cereal yield and quality. In: Crop Responses and Adaptations to Temperature Stress. (ed A.S. Basra) 243–291 (Food Products, 2001).

[40] Sakata T, Takahashi H, Nishiyama I, Higahsitani A (2000). Effects of high temperature on the development of pollen mother cells and microspores in barley *Hordeum vulgare* L. J.Plant Res..

[41] Saini HS, Sedgley M, Aspinall D (1984). Developmental anatomy in wheat of male sterility induced by heat stress, water deficit or abscisic acid. Aust. J. Plant Physiol..

[42] Imin N, Kerim T, Weinman JJ, Rolfe BG (2006). Low temperature treatment at the young microspore stage induces protein changes in rice anthers. Mol. Cell. Proteomics.

[43] Saini HS (1997). Effect of water stress on male gametophyte development in plants. Sex.Plant Reprod..

[44] Sheoran IS, Saini HS (1996). Drought induced male sterility in rice: changes in carbohydrate levels and enzyme activities associated with the inhibition of starch accumulation in pollen. Sex. Plant Reprod..

[45] Dorian S, Lalonde S, Saini HS (1996). Induction of male sterility in wheat by meiotic stage water deficit is preceded by a decline in invertase activity and changes in carbohydrate metabolism in anthers. Plant Physiol..

[46] Datta R, Chamusco KC, Chourey PS (2002). Starch biosynthesis during pollen maturation is associated with altered patterns of gene expression in maize. Plant Physiol..

[47] Goetz M (2001). Induction of male sterility in plants by metabolic engineering of the carbohydrate supply. Proc. Natl. Acad. Sci.USA.

[48] Castro AJ, Clément C (2007). Sucrose and starch catabolism in the anther of *Lilium* during its development: a comparative study among the anther wall, locular fluid and microspore/pollen fractions. Planta.

[49] Dawson J (1999). Characterization and genetic mapping of a mutation (*ms35*) which prevents anther dehiscence in *Arabidopsis thaliana* by affecting secondary wall thickening in the endothecium. New Phytol..

[50] Yang D, Seaton DD, Krahmer J, Halliday J (2016). Photoreceptor effects on plant biomass, resource allocation, and metabolic state. Proc. Natl. Acad. Sci. USA.

[51] Frank G (2009). Transcriptional profiling of maturing tomato (*Solanumlycopersicum* L.) microspores reveals the involvement of heat shock proteins, ROS scavengers, hormones, and sugars in the heat stress response. J. Exp. Bot..

[52] Honys D, Twell D (2004). Transcriptome analysis of haploid male gametophyte development in *Arabidopsis*. Genome Biol..

[53] Sheoran IS, Ross AR, Olson DJ, Sawhney VK (2007). Proteomic analysis of tomato (*Lycopersicon esculentum*) pollen. J. Exp. Bot..

[54] Volkov RA, Panchuk II, Schöffl F (2005). Small heat shock proteins are differentially regulated during pollen development and following heat stress in tobacco. Plant Mol.Biol..

[55] Yang KZ (2009). A mutation in *Thermo sensitive Male Sterile 1*, encoding a heat shock protein with DnaJ and PDI domains, leads to thermosensitive gametophytic male sterility in Arabidopsis. Plant J..

[56] Giorno F (2010). Developmental and heat stress-regulated expression of HsfA2 and small heat shock proteins in tomato anthers. J. Exp. Bot..

[57] Alexander MP (1969). Differential staining of aborted and nonaborted pollen. Stain Tech..

[58] Xu XH (2012). Identification of genes specifically or preferentially expressed in maize silk reveals similarity and diversity in transcript abundance of different dry stigmas. BMC genomics.

[59] Mortazavi, A., Williams, B. A., McCue, K., Schaeffer, L. & Wold, B. Mapping and quantifying mammalian transcriptomes by RNA-Seq. *Nat. methods* **5**, 621–628 (2008). 10.1038/nmeth.1226 PMC13303166 18516045

[60] Anders S, Huber W (2010). Differential expression analysis for sequence count data. Genome Biol..

[61] Du, Z., Zhou, X., Ling, Y., Zhang, Z. & Su, Z. agriGO: a GO analysis toolkit for the agricultural community. *Nucleic Acids Res*. **38**, W64–70 (2010). 10.1093/nar/gkq310 PMC2896167 20435677

