# Supplementary material for: The Rice Phytochrome Genes, PHYA and PHYB, Have Synergistic Effects on Anther Development and Pollen Viability
Source: Sci Rep. 2017 Jul 25;7:6439. doi: 10.1038/s41598-017-06909-2 (PMC5527001; doi:10.1038/s41598-017-06909-2)
Supplement: Supplementary file 1 — Supplementary Information [file 41598_2017_6909_MOESM1_ESM.pdf]

The Rice Phytochrome Genes, *PHYA* and *PHYB*, Have Synergistic Effects on Anther Development and Pollen Viability

Wei Sun[1,+], Xiao Hui Xu[2,+], Xingbo Lu[2], Lixia Xie[1], Bo Bai[1], Chongke Zheng[1], Hongwei Sun[2], Yanan He[1], Xian-zhi Xie[1, *]

[1]*Shandong Rice Research Institute, Shandong Academy of Agricultural Sciences, Ji'nan 250100, China*

[2]*Institute of Plant Protection, Shandong Academy of Agricultural Sciences, Shandong Key Laboratory of Plant Pathology, Ji'nan 250100, China*

[*]Corresponding author (Tel +86 531 8317 8661; email xzhxie2010@163.com)

[+]These authors contributed equally to this work.

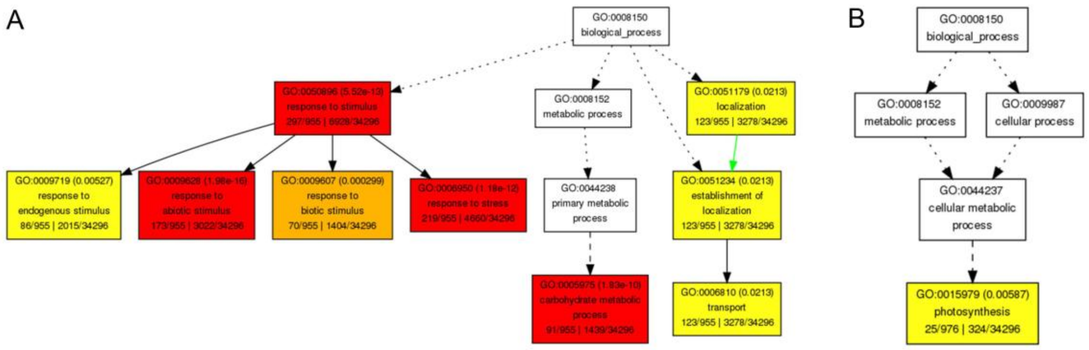

Figure S1. GO analysis of differentially expressed transcripts that specific to the *phyA phyB*/WT dataset. (A) Up-regulated transcripts in the *phyA phyB* mutant. (B) Down-regulated transcripts in the *phyA phyB* mutant.

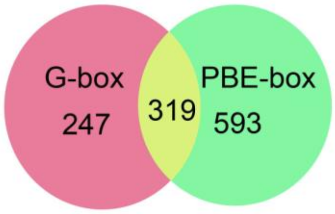

Figure S2. Distribution of G-box and PBE-box in the promoter regions of differentially expressed genes specific to the phyA phyB/WT dataset.
